# Targeted memory reactivation during sleep can induce forgetting of overlapping memories

**DOI:** 10.1101/lm.053594.122

**Published:** 2022-11

**Authors:** Bárður H. Joensen, Marcus O. Harrington, Sam C. Berens, Scott A. Cairney, M. Gareth Gaskell, Aidan J. Horner

**Affiliations:** 1 Department of Psychology, University of York, York YO10 5DD, United Kingdom; 2 Institute of Cognitive Neuroscience, University College London, London WC1N 3AZ, United Kingdom; 3 Institute of Neurology, University College London, London WC1N 3BG, United Kingdom; 4 School of Psychology, University of Sussex, Falmer BN1 9QH, United Kingdom; 5 York Biomedical Research Institute, University of York, York YO10 5NG, United Kingdom

## Abstract

Memory reactivation during sleep can shape new memories into a long-term form. Reactivation of memories can be induced via the delivery of auditory cues during sleep. Although this targeted memory reactivation (TMR) approach can strengthen newly acquired memories, research has tended to focus on single associative memories. It is less clear how TMR affects retention for overlapping associative memories. This is critical, given that repeated retrieval of overlapping associations during wake can lead to forgetting, a phenomenon known as retrieval-induced forgetting (RIF). We asked whether a similar pattern of forgetting occurs when TMR is used to cue reactivation of overlapping pairwise associations during sleep. Participants learned overlapping pairs—learned separately, interleaved with other unrelated pairs. During sleep, we cued a subset of overlapping pairs using TMR. While TMR increased retention for the first encoded pairs, memory decreased for the second encoded pairs. This pattern of retention was only present for pairs not tested prior to sleep. The results suggest that TMR can lead to forgetting, an effect similar to RIF during wake. However, this effect did not extend to memories that had been strengthened via retrieval prior to sleep. We therefore provide evidence for a reactivation-induced forgetting effect during sleep.

Stabilizing memories following learning is a critical function of system consolidation (Dudai et al. 2015). Theories of system consolidation have proposed that memory reinstatement during sleep constitutes one mechanism for transforming a newly acquired memory into a relatively stable, long-term form (Marr 1971; McClelland et al. 1995). This proposal is supported by rodent studies showing that patterns of neural activity, associated with information acquired during wake, are spontaneously reinstated during sleep (Wilson and McNaughton 1994), with reactivation predicting later retention (Dupret et al. 2010). Similarly, neuroimaging results in humans have shown that memory reactivation during non-rapid eye movement (NREM) sleep predicts later memory performance (Peigneux et al. 2004; Deuker et al. 2013).

Recently, studies have used a technique called targeted memory reactivation (TMR) to assess the role of memory reactivation during sleep. An early demonstration of this technique was reported by Rudoy et al. (2009), who used auditory stimuli to selectively increase retention for memories associated with the same sound during learning. In this study, participants learned a series of object–location pairs, with each pair presented alongside a characteristic sound (e.g., if the object was a “dog,” then the object would be presented alongside the sound of a dog barking). Rudoy et al. (2009) showed that presenting these same sounds to participants during NREM sleep improved retention relative to pairs associated with sounds not presented during sleep. This TMR effect has since been replicated using both naturalistic sounds (Van Dongen et al. 2012; Vargas et al. 2019) and spoken words (Cairney et al. 2017, 2018).

It has been proposed that memory reactivation during NREM sleep—or slow wave sleep (SWS) more specifically—helps strengthen the association between overlapping memories (Lewis and Durrant 2011). This proposal is motivated by evidence showing that participants are able to gain insight into logical problems (Wagner et al. 2004) and infer the relationship between objects embedded in a hierarchical structure (Ellenbogen et al. 2007) and overlapping pairs (Lau et al. 2010) following sleep. However, prior studies using TMR to assess the effect of memory reactivation have tended to focus on single pairs and, as such, have not been able to assess this prediction.

More recently, studies assessing the effect of TMR have begun to yield results in contrast to the proposal that reactivation during NREM sleep strengthens overlapping memories, with TMR differentially affecting memory for overlapping pairs depending on their relative encoding strength (Oyarzún et al. 2017) or which association was paired with high or low future reward (Antony et al. 2018). Notably, Oyarzún et al. (2017) used TMR to demonstrate that memory reactivation during NREM sleep can lead to differences in whether overlapping pairs are retained or forgotten. In this study, the encoding of two overlapping object–location pairs was manipulated such that the encoding of the pairs followed either immediately after each other or after a 3-h delay. The investigators reasoned that if memory strength decreases with time, then increasing the interval between encoding should lead to retroactive interference, such that the most recently encoded pair should be more readily retrieved. This was not assumed to be the case when the two pairs were encoded in short succession of each other. Consistent with this, Oyarzún et al. (2017) showed that presenting participants with auditory cues (previously presented during the encoding of the second pairs) during NREM sleep decreased memory for the first encoded pairs. However, this was only true when the encoding of the pairs was separated by a delay. When encoding followed immediately after each other, TMR instead resulted in an increase in memory for the first encoded pairs.

This study by Oyarzún et al. (2017) was the first to demonstrate that TMR can result in strengthening or weakening depending on a pair's strength relative to an overlapping pair. Critically though, this study only assessed memory for the first encoded pairs, not both the first and second encoded pairs. As such, we cannot know whether TMR leads to a different effect for each of the overlapping pairs or whether it results in a more general effect where memory for both pairs increases or decreases depending on the interval between encoding. Furthermore, the encoding of overlapping pairs with short intervals is susceptible to proactive interference (PI). For instance, Richter et al. (2016) provided evidence for PI occurring when participants learned two overlapping pairs in short succession of each other, with greater performance for the first relative to second encoded pairs. Thus, it is not only the relative strength of the first encoded pairs that is reduced when overlapping pairs are encoded at long delays. Rather, the strength of the second encoded pair is similarly diminished when encoding occurs at short intervals. In the current study, we only presented pairs at short intervals and so assume that memory strength will be greater for the first encoded than the second encoded pairs.

Interestingly, Schechtman et al. (2021a) found that sets of semantically related item–location pairings (e.g., six different cats in different locations) can be reactivated via TMR as effectively as single item–location pairs, suggesting that multiple overlapping memories can be reactivated in parallel without any differential effect in accuracy. However, what is critical in this study is that item–location pairs were learned to criterion, specifically to minimize differences in memory strength and within-set interactions. Similarly, Vargas et al. (2019) showed that overlapping item–location pairings associated with sound cues presented during sleep were remembered better than those associated with sounds not presented. However, item–location pairs were again learned to criterion prior to sleep in this study. We propose that when overlapping pairs differ in memory strength, it is possible that presenting a sound cue (associated with the overlapping pairs) during sleep results in greater reactivation of whichever pair is most strongly represented in memory. This point is critical given evidence showing that selective retrievals of a target association during wake can lead to forgetting of the nonretrieved, overlapping pair. For instance, Anderson et al. (1994) demonstrated that after encoding category–exemplar associations (e.g., “fruit: apple” vs. “fruit: pear”), repeated retrievals of selected associations decreased memory for exemplars not probed during retrieval, a phenomenon known as retrieval-induced forgetting (RIF). Wimber et al. (2015) more recently used fMRI to demonstrate that selective retrievals can lead to suppression of neural representations of overlapping, nonretrieved pairs, with the level of suppression predicting forgetting.

Based on the assumption that reactivation during sleep is sensitive to differences in memory strength, we reasoned that the increases in retention for first encoded pairs reported in Oyarzún et al. (2017)—when overlapping pairs were learned in short succession of each other—may reflect a RIF-like process (akin to those seen during wake) that also results in decreases in memory for second encoded pairs. This is because past research has shown that PI occurs when overlapping pairs are learned at short intervals (Richter et al. 2016), and presenting sound cues during sleep may, under these conditions, be more prone to initiate reactivation of the first/strongly encoded pairs and in turn decrease retention for the second/weakly encoded pairs.

To test this prediction, we had participants learn a series of object–location–person triplets, consisting of two pairs (e.g., “David Beckham–bicycle” and “castle–bicycle”) that shared an overlapping item (“bicycle”) (see Fig. 1). Critically, pairs from the same triplet were learned separately, such that the encoding of one pair from a given triplet was interleaved with the encoding of pairs from other triplets. Separating the encoding of pairs from the same triplet allowed us to manipulate the relative strength of the pairs depending on the encoding order (as a function of PI). To minimize any attempt to integrate across pairs from the same triplet, as this is known to diminish PI (Richter et al. 2016) and to act as a boundary condition for RIF (Anderson and McCulloch 1999), participants were not made aware of the overlapping nature of the pairs. We have previously shown that overlapping pairs under these encoding conditions do not show signs of integration, either immediately after encoding (Horner and Burgess 2014) or after a delay (Joensen et al. 2020).

**Figure 1.**
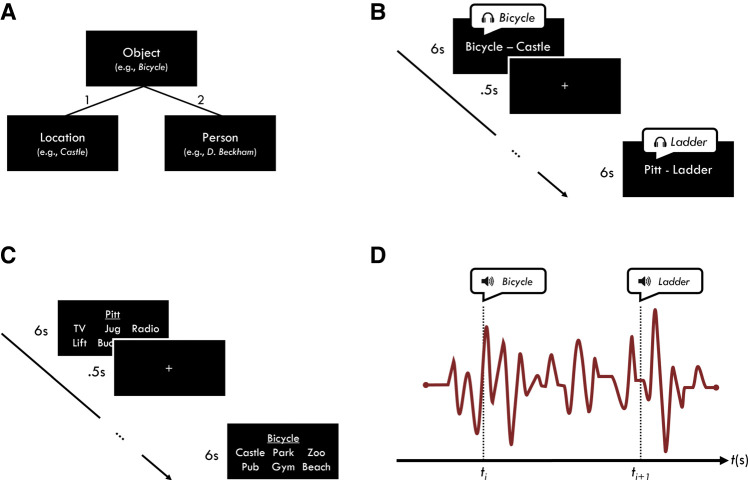
(*A*) Triplets. Three-item triplet with example encoding order (nos. 1 and 2). (*B*) Encoding. Participants learned two word pairs from 60 triplets (120 pairs in total). Each pair remained on screen for 6 sec and was preceded by a 500-msec fixation and followed by a 500-msec blank screen. The spoken object word was presented alongside the word pair, 1 sec after the trial onset, and 1 sec before the trial offset. The encoding phase was split into two blocks of 60 trials, with one pair from each triplet presented during each block. (*C*) Test. Participants were presented with a single cue and required to retrieve one of the other items from the same triplet from among five foils (items of the same type from other triplets; e.g., if the target word was “castle,” then the five foils would be other randomly selected locations from other triplets) within 6 sec. Each test trial was preceded by a 500-msec fixation and followed by a 500-msec blank screen. (*D*) TMR/sleep. Spoken words associated with half of the learned triplets were repeatedly presented via a speaker at 5-sec intervals (±300-msec random jitter). TMR began once participants entered SWS and was paused if participants showed signs of arousal/awakening or moved into a different sleep stage.

During encoding, each pair from the same triplet was presented alongside a triplet-specific spoken word. For instance, if participants studied the pairs “David Beckham–bicycle” and “castle–bicycle,” the word “bicycle” was presented auditorily alongside both word pair presentations. We then presented a subset of these spoken words to participants during overnight NREM sleep (specifically SWS) to assess the effect of TMR on retention for the first and second encoded pairs.

Alongside our prediction that TMR may facilitate retention for the first/strongly encoded pairs while decreasing retention for the second/weakly encoded pairs, we were also interested in the effect of retrieval prior to sleep (and TMR). Retrieval practice is known to promote long-term memory retention (Roediger and Karpicke 2006), and one proposal holds that this is because retrieval results in a high degree of memory strengthening. This increase in memory strength allows retrieved memories to remain above retrieval thresholds for prolonged periods of time (Kornell et al. 2011). Drawing from this proposal, Bäuml et al. (2014) suggested that if retrieved items are strengthened to such a high degree in the absence of sleep, then any further sleep-induced consolidation processes will only have, at best, a small effect on later memory performance. Antony et al. (2017) more recently proposed that retrieval itself may act as a rapid consolidation event. Although this account focuses on repeated retrievals rather than testing itself, it similarly suggests that sleep-mediated consolidation effects may be less visible for memories tested prior to sleep.

If true, these proposals suggest that testing prior to sleep may reduce any TMR-induced memory effects seen following sleep. To test this, half of the learned pairs were tested prior to sleep (and TMR). Our experimental design therefore allowed us to assess (1) the effect of TMR on retention for first/strongly versus second/weakly encoded overlapping pairs and (2) the effect of testing prior to sleep in a factorial design. Using this design, we found evidence for increased retention for strongly encoded pairs in the TMR relative to the non-TMR (NTMR) condition and decreased retention for weakly encoded pairs in the TMR relative to the NTMR condition. However, this pattern of increased and decreased retention was only present in pairs not tested prior to sleep, suggesting that testing diminished the TMR-induced modulation of memory.

## Results

### Behavior

#### Integration of overlapping pairs

Independence, or the absence of integration among overlapping pairs, is a boundary condition for PI (Richter et al. 2016) and RIF (Anderson and McCulloch 1999). We have shown previously that overlapping pairs encoded under conditions similar to those used here do not show evidence of integration (Horner and Burgess 2014; Joensen et al. 2020). To demonstrate that this also extends to the current paradigm, we computed a retrieval dependency measure that has previously been used to infer the presence (or absence) of integration (Horner and Burgess 2013). Retrieval dependency estimates the statistical relationship between retrieval successes of pairs from the same object–location–person triplet. For example, if a participant is cued with “bicycle” and correctly retrieves “David Beckham,” is the participant then more likely to retrieve “castle” when cued with “bicycle” on a separate retrieval trial? The retrieval dependency measure is zero when no dependency is present and greater than zero when there is dependency between retrievals within triplets.

Mean retrieval dependency and standard deviations at immediate (T1) and delayed (T2) tests (separately for the TMR and NTMR conditions) are presented in Table 1. First, we asked whether there was evidence for dependency across all pairs. A one-sample *t*-test comparing retrieval dependency (across all pairs) versus zero showed no evidence for dependency (*t*_(28)_ = 1.41, *P* = 0.17, *d* = 0.26). Next, to assess whether dependency differed (and/or changed) as a function of session or TMR status, we performed a 2 × 2 (T1 vs. T2 × TMR × NTMR) ANOVA on dependency. For this analysis, we only included estimates of dependency at T2 for pairs that were not previously tested at T1. The ANOVA revealed a significant effect of TMR (*F*_(1,28)_ = 5.79, *P* = 0.23, η_*p*_^2^ = 0.17), with less dependency (collapsed across T1 and T2) in the TMR relative to the NTMR (*t*_(28)_ = 2.41, *P* = 0.02, *d* = 0.45) condition. However, there was no evidence for a main effect of session (*F*_(1,28)_ = 0.34, *P* = 0.56, η_*p*_^2^ = 0.01) or interaction between session and overnight TMR (*F*_(1,28)_ = 0.07, *P* = 0.79, η_*p*_^2^ = 0.03), suggesting that dependency did not decrease or increase following sleep.

**Table 1.**
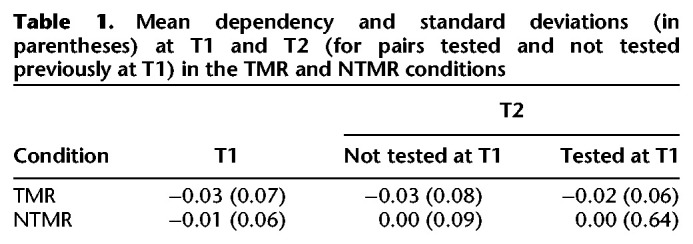
Mean dependency and standard deviations (in parentheses) at T1 and T2 (for pairs tested and not tested previously at T1) in the TMR and NTMR conditions

We also assessed whether dependency at T2 differed depending on whether the pairs had previously been tested at T1. To do this, we performed at 2 × 2 (tested vs. not tested × TMR vs. NTMR) ANOVA on dependency at T2. This ANOVA revealed no significant effects (*F*s < 0.19, *P*s > 0.66), but we note a trend for a significant effect of TMR (*F*_(1,28)_ = 3.57, *P* = 0.07, η_*p*_^2^ = 0.11).

As such, we show that overlapping pairs encoded under the current conditions do not show signs of integration, either immediately after encoding or after a period of sleep (and TMR). Interestingly, we did see an effect of TMR, suggesting that dependency (collapsed across pairs at T1 and not-tested pairs at T2) was significantly lower for pairs in the TMR compared with the NTMR condition. To examine whether this difference was driven by the presence of dependency in the NTMR condition, we assessed whether there was evidence for dependency in the TMR or NTMR condition (collapsed across pairs at T1 and not-tested pairs at T2). One-sample *t*-tests comparing dependency versus zero revealed no evidence of dependency for pairs in the NTMR condition (*t*_(28)_ = 0.55, *P* = 0.59, *d* = 0.10), but we saw evidence for antidependency for pairs in the TMR condition (*t*_(28)_ = 2.78, *P* = 0.01, *d* = 0.52). We tentatively suggest that this can be taken to imply that pairs in the TMR condition interfere with each other at retrieval, such that the successful retrieval of one pair decreases the likelihood of successfully retrieving the other pair from the same triplet. Although we were only able to measure dependency at retrieval, it is possible that this potential interference emerges at encoding or in the interval between encoding and retrieval, rather than retrieval per se.

#### Memory for first vs. second encoded pairs

Next, we asked whether the first encoded pairs were remembered better than the second encoded pairs (regardless of TMR), consistent with the presence of PI. Note that as each pair was tested in both directions (e.g., [1] cue: “David Beckham,” target: “bicycle” and [2] cue: “bicycle,” target: “David Beckham”), memory performance is equal to the average performance over the two retrieval trials (i.e., the proportion of correct retrievals across the two trials) for the first and second encoded pairs, respectively. Table 2 presents mean proportion correct and standard deviations for the first and second encoded pairs, separately for the TMR and NTMR condition, at T1 and T2.

**Table 2.**
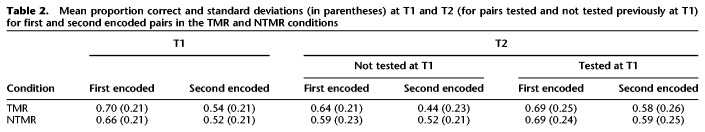
Mean proportion correct and standard deviations (in parentheses) at T1 and T2 (for pairs tested and not tested previously at T1) for first and second encoded pairs in the TMR and NTMR conditions

Table 2 suggests overall greater memory performance for the first relative to the second encoded pairs. Consistent with this, paired sample *t*-tests confirmed that memory performance was significantly greater for the first relative to the second encoded pairs at both T1 (collapsed across the TMR and NTMR conditions; *t*_(28)_ = 5.91, *P* < 0.001, *d* = 1.10) and T2 (collapsed across the TMR and NTMR and across the tested and not-tested conditions; *t*_(28)_ = 5.07, *P* < 0.001, *d* = 0.94).

#### Memory for first vs. second encoded pairs following TMR

We next asked whether this difference between the first and second encoded pairs was modulated by overnight TMR. To assess this, we performed a 2 × 2 × 2 (T1 vs. T2 × first vs. second encoded pair × TMR vs. NTMR) ANOVA on memory performance. For this analysis, we only included performance at T2 for pairs that were not tested previously at T1. The ANOVA revealed a significant three-way interaction between session, encoding order, and TMR (*F*_(1,28)_ = 5.11, *P* = 0.03, η_*p*_^2^ = 0.16). Main effects of encoding order (*F*_(1,28)_ = 35.93, *P* < 0.001, η_*p*_^2^ = 0.56), session (*F*_(1,28)_ = 16.79, *P* < 0.001, η_*p*_^2^ = 0.38), lower-order interactions between TMR and session (*F*_(1,28)_ = 12.43, *P* < 0.01, η_*p*_^2^ = 0.31), and TMR and encoding (*F*_(1,28)_ = 7.44, *P* = 0.01, η_*p*_^2^ = 0.21) were also present.

**Figure 2.**
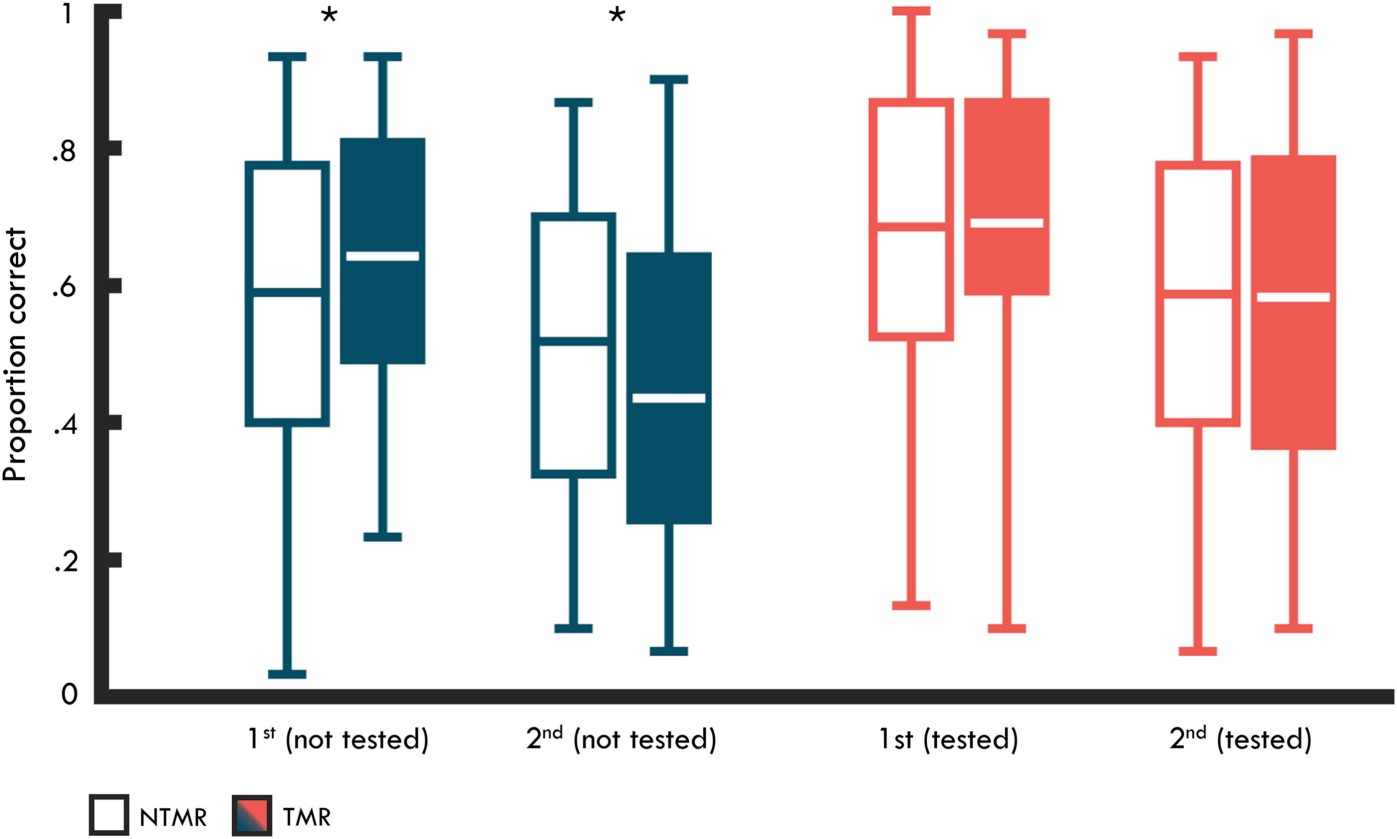
Mean proportion correct at T2 (for pairs not tested previously at T1 and tested previously at T1) for first and second encoded pairs in the TMR (filled) and NTMR (unfilled) conditions. Lines in boxes represent mean performance in each condition. *Bottom* and *top* edges of boxes indicate the 25th and 75th percentiles, respectively. Whiskers represent the minimum and maximum data points. Note that for pairs tested previously at T1, proportion correct reflects memory performance after triplets were removed to equate performance at T1. (*) *P* < 0.05.

To interrogate the three-way interaction further, we performed 2 × 2 (first vs. second encoded pair × TMR vs. NTMR) ANOVAs separately for T1 and T2. At T2, the ANOVA revealed a significant two-way interaction between TMR and encoding order (*F*_(1,28)_ = 10.90, *P* < 0.01, η_*p*_^2^ = 0.28), with pairwise comparisons revealing that while retention for the first encoded pairs was significantly greater in the TMR relative to the NTMR condition (*t*_(28)_ = 2.13, *P* = 0.04, *d* = 0.40), memory for the second encoded pairs was significantly lower in the TMR compared with the NTMR condition (*t*_(28)_ = 3.53, *P* < 0.01, *d* = 0.66) (see Fig. 2). We therefore show that TMR can support memory for the first/strongly encoded pairs and decrease memory performance for the second/weakly encoded pair (relative to the NTMR condition). This latter finding suggests that TMR can be used to induce forgetting under specific experimental conditions (Schechtman et al. 2020, 2021b).

Importantly, a 2 × 2 ANOVA at T1 revealed no interaction between encoding order and TMR (*F*_(1,28)_ = 0.21, *P* = 0.65, η_*p*_^2^ < 0.01), demonstrating that the TMR effect for first and second encoded pairs was not seen prior to sleep. However, the ANOVA at T1 revealed a main effect of TMR on memory performance (*F*_(1,26)_ = 75.18, *P* < 0.001, η_*p*_^2^ = 0.73), with memory for pairs in the TMR condition, irrespective of encoding order, being greater than in the NTMR condition prior to sleep. This is because triplets tested at T1 were assigned to the TMR and NTMR conditions by rank-ordering performance in a descending (and interleaved) order (i.e., first, third … *N* − 1 vs. second, fourth … *N*), and this procedure was not counterbalanced across participants (caused by an error during data collection). This means that the triplet with the highest level of accuracy was consistently assigned to the TMR condition, and reversely, the triplet with the lowest level of accuracy was consistently assigned to the NTMR condition. Note that the triplets not tested at T1 were assigned to the TMR and NTMR conditions in a randomized manner, and as such it is unlikely that the TMR effect seen at T2 (for not-tested pairs) is driven by differences in performance at T1.

However, for completeness, we report two separate ANCOVAs comparing differences in memory performance between first and second encoded pairs at T2 as a function of TMR, where we controlled for memory differences at T1. Consistent with the results reported above, the ANCOVA revealed a significant effect of TMR for the second encoded pairs (*F*_(1,55)_ = 9.74 *P* < 0.01, η_*p*_^2^ = 0.15), with lower memory performance for pairs in the TMR relative to the NTMR condition. However, the ANCOVA revealed no effect of TMR for first encoded pairs at T2 (*F*_(1,55)_ = 0.30, *P* = 0.59, η_*p*_^2^ < 0.01).

Thus, we show that TMR during overnight SWS is associated with decreases in memory performance for the second encoded pairs relative to pairs in the NTMR condition, even when controlling for any differences at T1. In contrast, after controlling for differences in performance at T1, we did not find evidence to show that TMR supports memory performance for the first encoded pairs. Interestingly, research on RIF has consistently shown that repeated retrieval is associated with decreases in memory performance for the nonretrieved pair, while increases in performance for the target memory are less consistently seen (Anderson et al. 1994; Wimber et al. 2015). These findings are broadly consistent with the TMR effects observed here. We return to these findings in the Discussion.

#### Testing effect on memory following TMR

Next, we assessed whether the effect of TMR at T2 differed between triplets tested and not tested at T1. To do this, we conducted a 2 × 2 × 2 (tested vs. not tested × first vs. second encoded pair × TMR vs. NTMR) ANOVA for memory performance at T2. This analysis includes both triplets not tested at T1 and triplets tested at T1 (where we saw that memory was greater for triplets in the TMR relative to the NTMR condition prior to sleep). As such, for tested triplets, we removed the lowest-performing triplet in the NTMR condition and the triplet closest to mean performance in the TMR condition to equate performance between the TMR and NTMR conditions at T1 (*t*_(28)_ = 0.38, *P* = 0.71, *d* = 0.07). Accordingly, for this analysis, the number of triplets per condition was (1) 14 tested triplets in the TMR condition, (2) 14 tested triplets in the NTMR condition, (3) 15 not-tested triplets in the TMR condition, and (4) 15 not-tested triplets in the NTMR condition. Note that the critical three-way interaction between session, encoding order, and TMR from the previous subsection remains significant following the removal of these pairs (*F*_(1,28)_ = 4.79, *P* = 0.04, η_*p*_^2^ = 0.15).

The 2 × 2 × 2 ANOVA revealed a significant three-way interaction between prior testing, TMR, and encoding order (*F*_(1,28)_ = 6.11, *P* = 0.02, η_*p*_^2^ = 0.18). In contrast to triplets not tested prior to sleep (see analyses above in “Memory for First vs. Second Encoded Pairs Following TMR”), for tested triplets, we saw no evidence for TMR differentially affecting memory for first and second encoded pairs at T2 (*F*_(1,28)_ = 0.02, *P* = 0.86, η_*p*_^2^ < 0.01) (see Fig. 2). Paired-sample *t*-tests confirmed that memory did not differ between the TMR and NTMR conditions for either the first (*t*_(28)_ = 0.74, *P* = 0.47, *d* = 0.14) or second (*t*_(28)_ = 0.32, *P* = 0.75, *d* = 0.06) encoded pairs when these pairs had been tested prior to sleep. The ANOVA also revealed a main effect of encoding order (*F*_(1,28)_ = 27.11, *P* < 0.001, η_*p*_^2^ = 0.49) and prior testing (*F*_(1,28)_ = 47.11, *P* < 0.001, η_*p*_^2^ = 0.63), with greater memory for triplets tested prior to sleep.

Thus, we see evidence that the effect of overnight TMR appears to be distinct for pairs not tested prior to sleep. To illustrate this further, we repeated the initial 2 × 2 × 2 (T1 vs. T2 × first vs. second encoded pair × TMR vs. NTMR) ANOVA (see analyses above in “Memory for First vs. Second Encoded Pairs Following TMR”), but for T2 we now only included tested triplets (as compared with not-tested triplets above in “Memory for First vs. Second Encoded Pairs Following TMR”). This ANOVA again revealed a significant effect of encoding order (*F*_(1,28)_ = 29.62, *P* < 0.001, η_*p*_^2^ = 0.51). However, consistent with the observation that the TMR effect does not extend to tested pairs, we saw no evidence for a three-way interaction between session, TMR, and encoding order (*F*_(1,28)_ = 0.45, *P* = 0.51, η_*p*_^2^ = 0.02). Note that for this ANOVA we removed triplets to equate performance at T1, but the ANOVA also failed to show evidence for a three-way interaction in the absence of doing this (*F*_(1,28)_ = 0.03, *P* = 0.87, η_*p*_^2^ < 0.01).

Interestingly, the ANOVA did reveal a significant interaction between session and encoding order (*F*_(1,28)_ = 6.04, *P* < 0.01, η_*p*_^2^ = 0.18). While performance for the first encoded pairs did not differ between T1 and T2 (*t*_(28)_ = 0.09, *P* = 0.93, *d* = 0.02), testing at T1 was associated with an increase in memory for the second encoded pairs between T1 and T2 (*t*_(28)_ = 2.54, *P* = 0.01, *d* = 0.47). This suggests that testing prior to sleep primarily contributed to strengthening the second/weakly encoded pairs, and this might be why the TMR-induced effect for not-tested triplets was not seen for tested triplets. We return to these findings in the Discussion.

### EEG

During the TMR period, participants were presented with sounds associated with triplets not tested and tested at T1, as well as control sounds that they had not encountered during learning. In addition to monitoring participants’ sleep, we also recorded EEG signals from eight scalp electrodes during this period. Here, we wanted to assess changes in neural oscillations when participants were presented with sounds associated with triplets learned at encoding as well as control sounds. Note that the current experiment was designed to maximize a behavioral effect, and as such the analyses of neural oscillations are exploratory and we do not present any predictions about time or frequency ranges that may reflect underlying consolidation processes.

**Figure 3.**
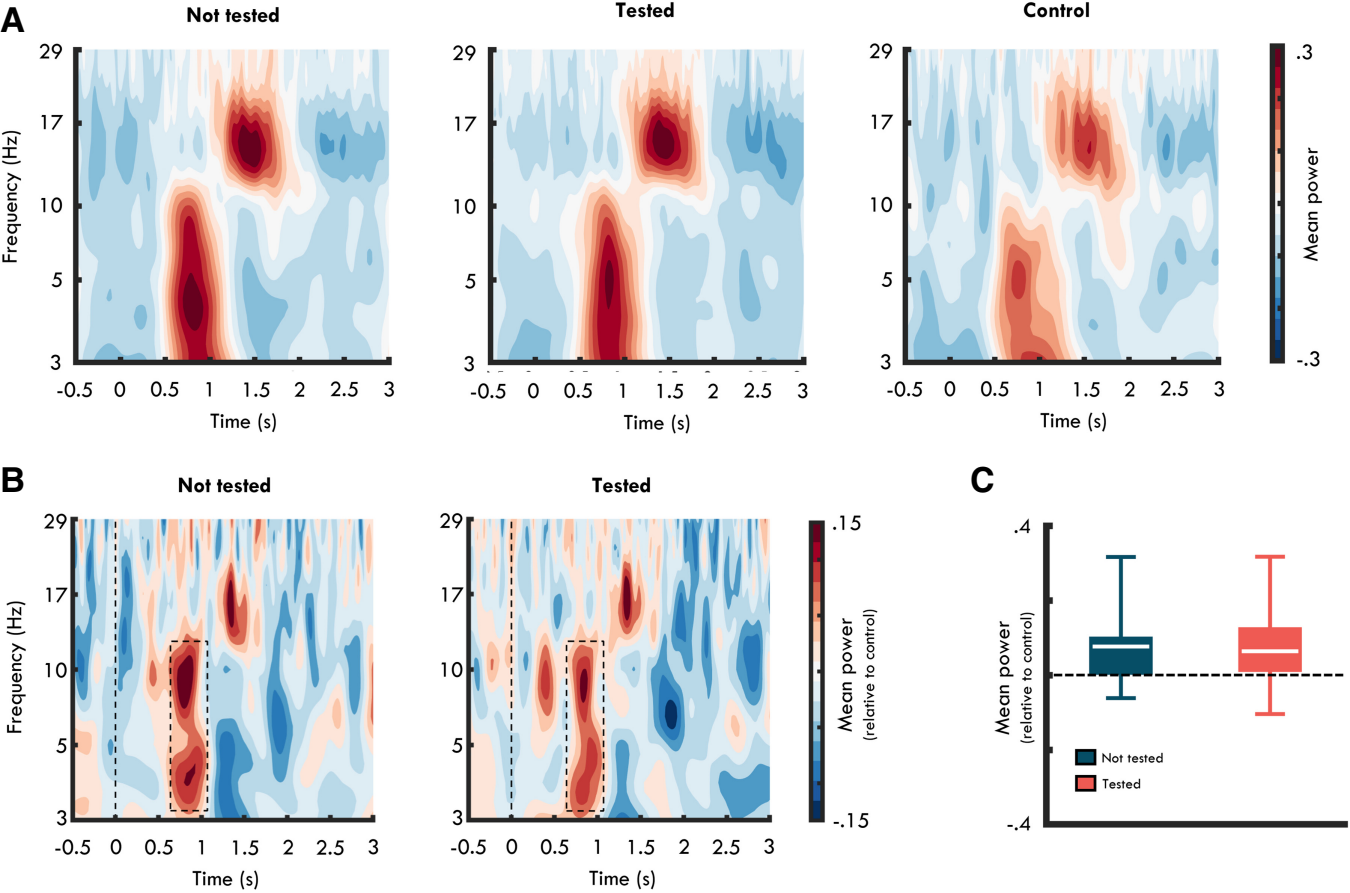
(*A*) Time–frequency power spectrogram following presentations of sounds associated with triplets not tested at T1 (*left*), triplets tested at T1 (*middle*), and control sounds (*right*). (*B*) Time–frequency power spectrogram following presentations of sounds associated with triplets not tested at T1 (*left*) and triplets tested at T1 (*right*) relative to control sounds. (*C*) Mean power at 3–12 Hz between 600 and 1100 msec (dashed boxes in *A*) following presentations of sounds associated with triplets not tested (blue) and tested (orange) at T1 relative to control sounds. Lines in boxes represent mean power in each condition. *Bottom* and *top* edges of boxes indicate the 25th and 75th percentiles, respectively. Whiskers represent minimum and maximum data points.

A Monte-Carlo cluster analysis on time–frequency power comparing sound presentations associated with not-tested triplets, tested triplets, and control sounds revealed a cluster at 3–12 Hz between 600 and 1100 msec following sound onset where the three conditions differed significantly (*F* = 6.25, *P* < 0.001, η_*p*_^2^ = 0.13) (see Fig. 3). Pairwise comparisons of power averaged over 3–12 Hz from 600 to 1100 msec revealed that oscillatory power was greater when participants were presented with sounds associated with triplets not tested (*t*_(28)_ = 4.72, *P* < 0.001, *d* = 0.88) and tested at T1 (*t*_(28)_ = 3.63, *P* < 0.01, *d* = 0.68) relative to control sounds. Although these findings are broadly consistent with evidence showing that TMR is associated with increases in theta power for experimental versus control sounds (Schreiner et al. 2015; Schreiner and Rasch 2015) as well as sound cues associated with remembered versus forgotten memories (Lehmann et al. 2016; Göldi et al. 2019), we also saw evidence for differences in alpha power (8–12 Hz). Although numerical differences (both positive and negative) in this frequency range have previously been reported (Schreiner et al. 2015; Cairney et al. 2018), these differences were not statistically significant, as is the case in the present study.

Activity in the theta band has also been associated with TMR-induced changes in memory (Schreiner and Rasch 2015; Schreiner et al. 2015; Oyarzún et al. 2017). Given that we saw a TMR effect for not-tested triplets but not for tested triplets, it is possible that power at 3–12 Hz may differ between the presentation of sound cues associated with not-tested and tested triplets. Despite this possibility, there was no difference in power from 600 to 1100 msec between 3 and 12 Hz when comparing sound presentations associated with tested and not-tested triplets (*t*_(28)_ = 0.68, *P* = 0.51, *d* = 0.13). This was also the case if we constrained this comparison to a conventional theta frequency range between 3 and 7 Hz in the same time window (*t*_(28)_ = 0.46, *P* = 0.65, *d* = 0.09). Theta power during TMR was therefore similar for not-tested and tested triplets despite the clear behavioral difference between these conditions.

## Discussion

We show that TMR can lead to both increases and decreases in retention for overlapping pairs, depending on their encoding order. After participants learned overlapping pairs prior to sleep, we saw that retention for the first encoded pairs increased in the TMR relative to the NTMR condition (but see below), while memory performance for the second encoded pairs decreased as a function of overnight TMR. We therefore present novel evidence for both increased and decreased retention (i.e., forgetting) for overlapping pairs when presented with a common sound cue during SWS. We tentatively refer to this as reactivation-induced forgetting and suggest that TMR may be useful for inducing forgetting, even for memories that have not been intentionally “tagged” for forgetting prior to sleep (Schechtman et al. 2020) or those paired with a forgetting tone (Simon et al. 2018).

Prior to sleep, memory performance for the first encoded pairs was greater than that for the second encoded pairs. If greater performance relates to greater associative strength, then it is possible that TMR induces greater reactivation of the first/strongly encoded pairs. We also saw evidence for antidependency for pairs in the TMR condition (collapsed across pairs at T1 and not-tested pairs at T2). The presence of antidependency in the TMR condition may increase the likelihood of greater reactivation for the first/strongly encoded relative to the second/weakly encoded pairs. However, if antidependency is a marker of interference between overlapping pairs, the current results cannot distinguish between whether interference is driving the RIF-like effect caused by TMR or whether TMR is potentially increasing interference. Further work is needed to assess these possibilities, but we note that direct competition is not a prerequisite for RIF. As such, we believe that it is likely that differences in associative strength, irrespective of any evidence for antidependency, may increase the possibility of greater reactivation for the strong relative to the weakly encoded pairs. This may in turn support retention for the first encoded pairs and decrease memory for the second encoded pairs, in a manner similar to RIF effects seen during wake. Here, we saw evidence for decreased memory performance for the second encoded pairs following TMR. While we also saw evidence for better retention for the first encoded pairs in the TMR relative to the NTMR condition, this effect was not seen when we controlled for differences in memory prior to sleep. However, it is possible that any memory benefits become more apparent over time, as the effects of TMR do not always arise immediately following sleep (Cairney et al. 2018; Rakowska et al. 2021).

Although forgetting seems disadvantageous, RIF is thought to serve an adaptive purpose that resolves interference between overlapping memories and mitigates further forgetting due to interference (Anderson 2003). In this context, our results may reflect a similarly adaptive process where TMR minimizes interference by weakening overlapping memories based on their relative strength (or behavioral relevance more generally). As such, memory reactivation during sleep may weaken noisy or irrelevant information to avoid our memory system becoming oversaturated or unreliable (Tononi and Cirelli 2006; Poe 2017).

RIF has typically been associated with a top-down, inhibitory mechanism (Anderson 2003; Wimber et al. 2008) where it is the inhibitory process engaged to overcome interference that produces forgetting, rather than interference itself (Anderson 2003). Consistent with this, Wimber et al. (2015) showed that suppression of neural representations of the overlapping but nonretrieved pairs during selective retrievals was predicted by activity in prefrontal regions associated with inhibitory control. Furthermore, Kuhl et al. (2008) demonstrated that activity in these regions decreases as overlapping pairs are forgotten but is re-engaged when a previously selected against memory is required to be retrieved. Critically, the evidence presented here, alongside those previously reported by Oyarzún et al. (2017), suggests that forgetting following repeated memory reactivation can also occur during sleep. Thus, the proposed inhibitory mechanism is relatively automatic and can be induced when the brain is in a different state (i.e., SWS) from wake.

Interestingly, Oyarzún et al. (2017) demonstrated that their TMR-induced memory effect was eliminated when sound cues were presented to awake participants. As such, it is possible that the effects reported in the current experiment are sleep-specific and perhaps relate to proposed differences in sleep and wake retrieval states (Hasselmo and Giocomo 2006). Instead, for a similar effect to be observed during wake, it is likely that participants would need to be instructed to repeatedly retrieve the first encoded pairs, but not the second encoded pairs, akin to RIF paradigms.

Computational accounts (Norman et al. 2006, 2007) have suggested that RIF is due to differences in activation at retrieval, such that forgetting occurs when levels of activation differ between a target and nontarget memory. According to these accounts, pairs that are only moderately reactivated will tend to be weakened, whereas those that are strongly activated will be strengthened. In this sense, forgetting can occur without an explicit inhibitory process. This proposal may account for our findings under the assumption that TMR is more likely to lead to lower levels of activation for the second/weakly encoded pairs relative to the first/strongly encoded pairs. Manipulating the delay between encoding (as in Oyarzún et al. 2017) and testing (as in the current study) both pairs, alongside multivariate measures that assess reactivation during TMR (Belal et al. 2018; Cairney et al. 2018; Schreiner et al. 2018, 2021; Schechtman et al. 2022) could provide evidence for this prediction.

Here we assume that the decrease in memory for the second encoded pairs in the TMR relative to the NTMR condition is due to the weakening of these pairs. However, given that both the first and second encoded pairs are associated with a common item (and sound), decreases in performance could be accounted for by a process in which an increase in memory for the first encoded pair blocks retrieval for the second encoded pair. This blocking account (McGeoch 1942) assumes that the likelihood of retrieving an associate is determined by an item's relative strength to a cue. Hence, when one pair's relative strength is increased, the probability of retrieving an overlapping pair will decrease. As such, the strengthened pair will block the participants’ ability to retrieve any overlapping pair. Although we saw that TMR supports retention for the first encoded pairs, this effect did not persist when controlling for differences in memory (for unrelated pairs) prior to sleep. In contrast, the TMR-induced decrease in memory for the second encoded pairs persisted, even when controlling for any difference in presleep performance. Prior work has also shown that weakening of the nonretrieved pairs can be seen in the absence of any facilitation for target pairs (Anderson et al. 2000; Wimber et al. 2015) and, critically, that RIF is cue-independent, such that forgetting extends to items related to, but not directly associated with, the retrieval cue (Anderson and Spellman 1995). As such, decreases in memory performance for the second encoded pairs are more likely to be accounted for by a mechanism where these pairs are weakened as a function of overnight TMR. Finding a similar decrease in performance for the second encoded pairs in the TMR relative to the NTMR condition using independent probes at test—as in the RIF literature (Anderson and Spellman 1995; Hulbert et al. 2012)—would provide stronger evidence for this conclusion.

The TMR effect was only seen for pairs that were not tested prior to sleep. Although it is not entirely clear why this effect does not extend to tested pairs, one proposal holds that testing increases the strength of a memory to such an extent that it stays above retrieval thresholds over prolonged intervals (Kornell et al. 2011). Drawing from this, Bäuml et al. (2014) showed that sleep supports retention for memories subject to restudy, but not those tested prior to sleep (see also Ashton et al. 2022), suggesting that increases in memory strength afforded by testing may mitigate the role of sleep in memory consolidation. This may account for why some previous studies have only reported TMR-induced facilitations for pairs retrieved with low accuracy prior to sleep (Creery et al. 2015; Cairney et al. 2016). In the current case, we saw no TMR-induced strengthening or weakening of the second/weakly encoded pairs when these were tested prior to sleep. It is possible that prior testing in this instance increased memory strength such that even comparatively weak pairs became insensitive to any TMR-induced consolidation effects. Importantly, most past work assessing the effect of TMR has tended to focus on memories tested before sleep. Our results suggest that the effects reported in prior work may be reduced by prior testing.

Interestingly, when we compared memory for the second encoded pairs across T1 and T2, we saw that performance increased across testing sessions when these pairs had been tested prior to sleep. This is in contrast to not-tested pairs, where memory was lower at T2 relative to T1, irrespective of encoding order or TMR status. Although we still saw that memory was greater for the first relative to the second encoded pairs at T2, this finding suggests that testing prior to sleep may play a greater role in supporting memory for the second/weakly encoded pairs relative to the first/strongly encoded pairs. While there is evidence to show that testing can minimize PI (Szpunar et al. 2008; Nunen and Weinstein 2012), this is typically in reference to reducing the buildup of interference during encoding and not in the interval between encoding and retrieval, as in the current case. As such, we can only speculate what the mechanism underlying the increase in memory performance is, but it is interesting that this increase was only seen for the second encoded pairs—the same pairs where we saw that performance decreased following TMR when triplets were not tested before sleep. This may account for why we failed to see evidence for a TMR effect for pairs tested prior to sleep.

Evidence for increased theta activity following the presentation of sound cues during sleep has led to the proposal that activity in this frequency band may play an important role in sleep-related consolidation. Notably, it has been suggested that increased theta activity during sleep may signal memory reinstatement (Schreiner and Rasch 2015; Göldi et al. 2019). Results from our EEG time–frequency analysis showed that the presentation of sound cues related to pairs learned prior to sleep was associated with increased oscillatory power across the theta and alpha bands. This is in line with suggestions that activity in the theta band may be associated with the reactivation of memories acquired during wake (Schreiner and Rasch 2015; Göldi et al. 2019). However, we should emphasize that because the same object sounds were used as control sounds across all participants, it is possible that undue perceptual differences may contribute to the EEG difference between sound cues associated with the learned triplets and control sounds.

Although activity in the theta band has also been associated with TMR-induced changes in memory, we found no evidence for a difference in oscillatory power between sound presentations associated with triplets not tested (where we saw a TMR-induced memory effect) and tested before sleep. However, it should be noted that the current experiment was not specifically designed to test for such effects. Previous work (Schreiner and Rasch 2015; Schreiner et al. 2015) assessing the relationship between changes in memory following TMR and activity in the theta band has tended to contrast sound presentations related to information that participants either did not retrieve prior to sleep but did following sleep (i.e., gains) or remembered both before and after sleep (i.e., hits) with information that they recalled before but not following sleep (i.e., losses). Here we are unable to perform this analysis—first because pairs in the condition where we saw a TMR effect were not tested prior to sleep, and second because we cannot clearly delineate between sound presentations associated with triplets that are retained or forgotten. Specifically, for those triplets that showed an effect of TMR, TMR was associated with both an increase in retention for the first encoded pairs and a decrease in retention for the second encoded pairs. As such, any oscillatory activity that follows TMR will not be uniquely predictive of retention or forgetting. Instead, we assessed whether oscillatory activity differed between triplets where TMR-induced changes in memory occurred (i.e., not-tested triplets) as compared with triplets where TMR did not produce any differences in memory (i.e., tested triplets). This contrast did not reveal any significant differences in oscillatory activity following sound presentation. Nevertheless, our findings add to an extending literature demonstrating that TMR during sleep is associated with an increase in theta activity, and that this increase in theta power may be related to memory reinstatement (Schreiner and Rasch 2015; Göldi et al. 2019).

In summary, we provide novel evidence for a TMR-induced effect during SWS that is akin to RIF effects seen during wake. We also show that the effect of TMR appears to extend only to memories not tested prior to sleep. The idea that selective retrieval during wake can lead to forgetting is well documented, and we suggest that a similar mechanism may be active during sleep in instances where overlapping memories differ in their relative strength.

## Materials and Methods

### Participants

Thirty-nine participants were recruited to take part in the experiment. Participants were recruited from the University of York student population and took part in exchange for course credit or monetary compensation. Written informed consent was obtained from all participants. Participants were informed that they were taking part in a memory/sleep experiment but were not made aware that TMR would be taking place during sleep. The experiment was approved by the Research Ethics Committee of the Department of Psychology of the University of York.

Seven participants who underwent less than two full rounds of TMR (i.e., each spoken word was presented less than twice during sleep) were excluded from the analyses. Note that prior studies assessing the effect of TMR (primarily during a nap session) on memory retention (Rudoy et al. 2009; Oyarzún et al. 2017; Cairney et al. 2018; Schechtman et al. 2021a) have typically used a less stringent exclusion criteria (i.e., less than one full round of TMR). However, as we were specifically interested in the effect of repeated memory reactivation during sleep, we opted for a more conservative criterion. This is why we chose to focus on overnight sleep rather than a nap session, as this would afford us more opportunities to administer TMR.

A further two participants were excluded due to them waking up and becoming aware that sound cues were being presented. This was assessed during debrief. In one instance, the participant was able to recall specific words being presented, and in the other case the participant reported being aware that sounds were being presented but could not remember the specific words. Last, one participant was excluded due to below-chance performance (<16.7%) during the presleep test session (T1). Therefore, a total of 29 participants (17 male and 12 female; M ± SD age = 20.97 yr ± 4.02 yr) was included in the analyses.

We estimated an effect size of *d* = 0.40 for detecting a TMR memory effect from previous published work (Van Dongen et al. 2012; Creery et al. 2015; Schreiner and Rasch 2015; Cairney et al. 2016, 2018; Ashton et al. 2018; Göldi et al. 2019; Vargas et al. 2019). Cohen's *d* was calculated from reported *t*-statistics divided by the square root of the sample size (Lakens 2013). Using G*Power (Faul et al. 2009) with α = 0.05, *n* = 29, and *d* = 0.40, we estimated that we could detect a significant effect of TMR, if one is present, at power = 0.55. Note though that eight out of the 10 studies used in this estimation have assessed TMR effects across a nap session lasting ∼90 min rather than overnight sleep (as in the current study). It is possible that this can underestimate the power of a TMR effect, as the limited duration of a nap session will constrain the number of times sound cues can be presented. Furthermore, the average sample size of the studies used to estimate the effect size was 21 (range = 12–30) as compared with 29 in the current study.

### Materials

The word stimuli consisted of 60 locations (e.g., “castle”), 60 famous people (e.g., “David Beckham”), and 60 common objects (e.g., “bicycle”). From these, 60 randomized location–person–object triplets were created for each participant.

The auditory stimuli consisted of 66 spoken words. These corresponded to the 60 object words used in the main word stimulus set (e.g., “bicycle”), in addition to six novel object words that were used as control sounds during the TMR phase. These six object words were independent of the main word stimulus set and were used as the control sounds across all participants. Spoken object words, rather than object sounds, were used to ensure greater congruency between the two stimulus sets. All the spoken words (M ± SD duration = 0.66 sec ± 0.15 sec) were recorded in the same, female voice and adjusted for perceived loudness using Adobe Audition (ver 3).

### Procedure

The experiment consisted of a single encoding phase, a single overnight sleep phase, and two test phases. Session 1 took place at approximately 9:30 p.m. (M = 9:36 p.m., range = 8:13 p.m.–10:18 p.m.) and consisted of a single encoding phase and a test phase (T1). Session 2 took place the following morning at approximately 7:30 a.m. (M = 7:27 a.m., range = 7:04 a.m.–7:54 a.m.) and consisted of a single test phase (T2).

The Pittsburgh Sleep Quality Index (Buysse et al. 1989) was administered prior to the encoding phase. This was done to assess for any difficulties in sleep up to 1 mo prior to the experiment. Sleep quality ratings indicated that participants had no current history of sleep difficulties (M ± SD = 4.55 ± 1.80).

#### Encoding

During encoding, participants were presented with specific word pairs from each of the 60 randomly generated triplets. Participants learned one pair per trial. All pairs were presented on a computer screen as words, with one item to the left and one to the right of fixation. The left/right assignment was randomly chosen on each trial. The pairs remained on screen for 6 sec. To encourage stronger and more elaborative encoding, participants were instructed to imagine the items interacting as vividly as possible for the full 6 sec. Each trial was preceded by a 500-msec fixation and followed by a 500-msec blank screen.

The encoding phase consisted of two blocks of 60 trials with one pair from each triplet presented during each block, making a total of 120 encoding trials. A break of 20 sec followed every 30 encoding trials. Within each block, the order of presentation was randomized. The encoding order for the triplets across the two blocks was location–object, person–object, or person–object and location–object. This order was pseudorandomized for each participant such that the encoding order for 30 randomly chosen triplets was location–object and person–object, and for the other 30 triplets was person–object and location–object. Note that the object was always the common overlapping item. We did not manipulate whether objects, locations, or people were the common item across triplets, given evidence that integration can occur when multiple objects are associated with a single location but not vice versa (Radvansky and Zacks 1991). The use of objects as the common item therefore decreased the probability of integration, increasing the likelihood of seeing a difference in memory between the first and second encoded pairs.

On each encoding trial, the spoken object word was presented twice via headphones. For instance, if a participant was presented with the pair “David Beckham–bicycle,” the spoken object word “bicycle” would be presented alongside this presentation 1 sec after the trial onset and 1 sec before the trial offset. The spoken words were presented twice to increase participants’ opportunity to associate the spoken word with the word pairs. We chose to present the spoken word at the onset and offset of each trial to ensure that participants had an opportunity to imagine the two items of each pair interacting in the absence of the spoken word.

#### Test

During the test phases (T1 and T2), participants performed a six-alternative forced-choice task. On each trial, a cue and six possible targets were presented simultaneously on screen. The cue was presented in the middle of the screen with six possible targets—one target and five foils from the same category as the target (e.g., if the target was “bicycle,” the five foils would be other randomly selected objects from other triplets)—presented in two rows of three below the cue. The position of the correct target item was randomly selected on each trial. Participants had 6 sec to respond with a key press. Missing responses (i.e., responses that fell outside the 6-sec response window) were treated as incorrect responses (M ± SD percentage of missing responses = 6.35 ± 5.40). Participants received no feedback on their memory performance.

Each triplet was tested with one of the cue–target pairs in both directions. These were presented across four blocks with a single pair from each triplet tested in each block. For T1, 30 out of the 60 possible triplets were tested, making a total of 120 trials. For T2, all 60 triplets were tested, making a total of 240 trials. A break of 20 sec would follow every 30 trials. Each trial was preceded by a 500-msec fixation and followed by a 500-msec blank screen.

#### TMR setup

The 30 triplets tested at T1 were rank-ordered by performance in descending order. Of these, 15 triplets were assigned in an interleaved manner (from most to least accurate in steps of two; i.e., first, third … *N* − 1) to the TMR condition, and the remaining 15 triplets were assigned to the NTMR condition. Of the 30 triplets not tested at T1, 15 triplets were randomly allocated to the TMR and NTMR conditions, respectively. The spoken object words associated with triplets assigned to the TMR condition were presented auditorily in a random order to participants during the sleep period. An additional six control object words, which participants had not encountered during encoding, were presented in an intermixed manner with the main auditory stimuli.

#### Sleep period

The sleep period began at approximately 11:00 p.m. and lasted ∼8 h (M ± SD = 7 h 55 min ± 21 min). Participants were left to sleep in a laboratory bedroom while their brain activity was monitored with polysomnography (PSG). An Embla N7000 PSG system with RemLogic software (ver 3.4) was used to monitor participants’ sleep. EEG scalp electrodes were attached according to the international 10–20 system at eight locations—frontal (F3 and F4), central (C3 and C4), parietal (P3 and P4), and occipital (O1 and O2)—with each referenced to electrodes on the contralateral mastoid (A1 and A2). A ground electrode was attached to the forehead. Left and right electro-oculography electrodes were also attached, in addition to electromyography electrodes at the bilateral mentalis and submentalis. All electrodes had an impedance of <10 kΩ and were unfiltered and digitally sampled at 200 Hz.

During sleep, the spoken words were presented via a speaker (mounted ∼1.5 m above the bed) that was connected to an amplifier in a separate control room. TMR was initiated when participants were in NREM sleep stage N3 (i.e., SWS), as identified online by the experimenter. We focused on SWS because it has been shown that this period of sleep provides a window for selectively strengthening memories via TMR (Rudoy et al. 2009; Fuentemilla et al. 2013; Cairney et al. 2017). The spoken words were presented in a randomized order at a sound intensity of ∼40 dB.

The presentation order was blocked such that all spoken words (including control sounds) were presented before any of the same words were presented again. This means that sounds (associated with both tested and not-tested triplets, as well as control sounds) were presented once during each block of TMR such that, across full rounds of TMR, each word was presented the same number of times (i.e., if the participants underwent three rounds of TMR, then each spoken word in the tested, not-tested, and control conditions was presented three times).

To avoid any auditory interference that may occur when sound cues are presented in rapid succession of each other (Schreiner et al. 2015), each sound presentation was separated by a 5-sec interval (±300-msec random jitter). TMR was continued for as long as participants were in SWS between sleep onset and approximately 4:00 a.m. (M ± SD number of full rounds of TMR = 7.34 ± 5.20). TMR was immediately paused if participants showed signs of arousal/awakening or moved into a different stage of sleep.

### Retrieval dependency

To estimate dependency, two 2 × 2 contingency tables were generated for each participant. These tables reflect the proportion of joint retrieval and nonretrieval between the person and location (e.g., “David Beckham” and “castle”) when cued by the common object (e.g., “bicycle”) and retrievals of the common object (e.g., “bicycle”) when cued by the person and location (e.g., “David Beckham” and “castle”) from the same triplet. The contingency tables were generated separately for the data and what we refer to as the independent model. The independent model assumes that pairs from a given triplet are retrieved independently of one another (e.g., if a participant is cued with “bicycle” and retrieves “David Beckham” successfully, then this does not predict the participant's ability to retrieve “castle” when cued with “bicycle” on a later retrieval trial). As such, the independent model serves as the lower bound that we can compare with the proportion of joint retrieval and nonretrieval in the observed data. As the proportion of joint retrieval and nonretrieval scales with accuracy, only comparisons between the data and independent model provide a meaningful estimate of dependency.

The contingency table for the independent model (Table 3) shows the predicted proportion of triplets that fall in the four cells, given a participant's overall level of performance, if retrievals of within-triplet pairs are assumed to be independent. For a given participant, the proportion of correct retrievals of, for instance, item B (e.g., “David Beckham”) when cued by A (e.g., “bicycle”) is denoted by *P*_*AB*_ (i.e., mean performance for B when cued by A across all triplets). For the independent model, when cued by A, the probability of (1) correctly retrieving both B and C (e.g., “castle”) across all triplets is equal to *P*_AB_*P*_AC_, (2) correctly retrieving B but not C is equal to *P*_AB_(1 − *P*_AC_), (3) correctly retrieving C but not B is equal to (1 − *P*_AB_)*P*_AC_, and (4) incorrectly retrieving both B and C is equal to (1 − *P*_AB_)(1 − *P*_AC_). We calculated the difference between joint retrievals and joint nonretrievals in the data and independent model for each participant and condition, averaged across the two contingency tables. We refer to this difference measure as “retrieval dependency,” where a value of zero denotes no evidence of retrieval dependency and values greater than zero denote evidence for retrieval dependency.

**Table 3.**
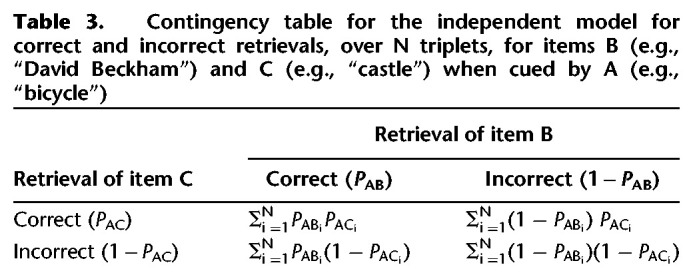
Contingency table for the independent model for correct and incorrect retrievals, over N triplets, for items B (e.g., “David Beckham”) and C (e.g., “castle”) when cued by A (e.g., “bicycle”)

### EEG analysis

For the EEG analysis, we first sleep-scored the continuous EEG data in accordance with the criteria of the American Academy of Sleep Medicine (Iber et al. 2007). See Table 4 for mean durations and standard deviations in minutes of sleep and percentages of sleep stages N1, N2, N3, and REM.

**Table 4.**
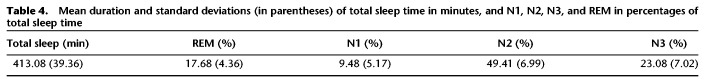
Mean duration and standard deviations (in parentheses) of total sleep time in minutes, and N1, N2, N3, and REM in percentages of total sleep time

Next, we removed all channels that did not maintain an impedance of <10 kΩ throughout the TMR period (M ± SD number of channels removed = 1.10 ± 1.37). As signal quality in occipital channels (O1 and O2) exceeded our impedance threshold in several participants, we omitted occipital channels for all participants from the analysis. We then segmented the continuous EEG data into epochs from −1000 to 3500 msec around the sound onset, and the data were high-pass-filtered at 0.1 Hz, notch-filtered at 48–52 Hz, and baseline-corrected with the first 500 msec immediately prior to the onset of the sound cue. Last, all epochs that did not continuously display N3/SWS throughout the entire epoch window were removed, the remaining epochs were manually screened, and those containing arousal and movement artifacts were additionally removed (M ± SD percentage of excluded epochs was 5.71 ± 11.61 for not-tested triplets, 6.24 ± 12.45 for tested triplets, and 5.58 ± 10.93for control sounds). There was no difference in the proportion of removed epochs across conditions (*F*_(2,84)_ = 0.03, *P* = 0.97, η_*p*_^2^ < 0.01).

Next, estimates of oscillatory power were obtained by convolving the EEG signal from each trial and channel with a five-cycle Morlet wavelet (Torrence and Compo 1998). Power values were obtained from 35 logarithmically spaced frequencies in the 3- to 30-Hz range. Power values between −1000 and −500 msec prior to and 3000 and 3500 msec after the sound onset were discarded after convolution (to avoid edge effects), and values between −500 and 3000 msec were *z*-transformed to give a measure of normalized power in each frequency and time step. Because we removed channels that exceeded our impedance threshold individually for each participant, oscillatory power was first averaged across all channels for each participant and then analyzed using a Monte-Carlo cluster permutation approach (number of permutations = 2000) (Maris and Oostenveld 2007) to detect clusters in time and frequency space where sound presentations associated with not-tested triplets, tested triplets, and control sounds differed. Clusters that produced a *P*-value of <0.05 were considered significant. For these clusters, *F*-values reflect the mean *F*-statistic within the cluster, and η_*p*_^2^ was used as the measure of the effect size (Lakens 2013). Note that as we averaged the signal across channels for each participant, the cluster permutation approach is not constrained by whether any effect is present in (a minimum of) two or more adjacent channels. Hence, the permutation approach only corrects for comparisons in time and frequency space.

### Data availability

All data and analyses are available on the Open Science Framework (https://osf.io/q2sfv).
